# Smooth deuterated cellulose films for the visualisation of adsorbed bio-macromolecules

**DOI:** 10.1038/srep36119

**Published:** 2016-10-31

**Authors:** Jielong Su, Vikram S. Raghuwanshi, Warwick Raverty, Christopher J. Garvey, Peter J. Holden, Marie Gillon, Stephen A. Holt, Rico Tabor, Warren Batchelor, Gil Garnier

**Affiliations:** 1 BioPRIA- Bioresource Processing Research Institute of Australia, Department of Chemical Engineering, Monash University, Clayton, Victoria, 3800, Australia; 2 Australian Nuclear Science and Technology Organisation (ANSTO) Locked Bag 2001, Kirrawee DC NSW 2232, Australia; 3 School of Chemistry, Monash University, Clayton, Victoria, 3800, Australia.

## Abstract

Novel thin and smooth deuterated cellulose films were synthesised to visualize adsorbed bio-macromolecules using contrast variation neutron reflectivity (NR) measurements. Incorporation of varying degrees of deuteration into cellulose was achieved by growing *Gluconacetobacter xylinus* in deuterated glycerol as carbon source dissolved in growth media containing D_2_O. The derivative of deuterated cellulose was prepared by trimethylsilylation(TMS) in ionic liquid(1-butyl-3-methylimidazolium chloride). The TMS derivative was dissolved in toluene for thin film preparation by spin-coating. The resulting film was regenerated into deuterated cellulose by exposure to acidic vapour. A common enzyme, horseradish peroxidase (HRP), was adsorbed from solution onto the deuterated cellulose films and visualized by NR. The scattering length density contrast of the deuterated cellulose enabled accurate visualization and quantification of the adsorbed HRP, which would have been impossible to achieve with non-deuterated cellulose. The procedure described enables preparing deuterated cellulose films that allows differentiation of cellulose and non-deuterated bio-macromolecules using NR.

Cellulose is a renewable biopolymer that can be produced in relatively pure form at low cost. It is, hydrophilic, biodegradable, biocompatible and easy to functionalize due to the six hydroxy groups on each of the anhydrocellobiose units that comprise the straight chain polymer. Apart from its traditional uses in the manufacture of textiles and paper, cellulose is finding new uses in bio-diagnostic devices and other functional surfaces that take advantage of its regular polysaccharide surface and well-characterised composition. Recently, the development of bioactive paper and the production of bioethanol from the cellulosic components of wood and agricultural residues have attracted wide interest. Paper has been engineered as a platform for low cost diagnostics in environmental and health applications where identification and/or quantification of specific biomolecules or chemical agents is essential^1^,^2^. Producing bioethanol from lignocellulosic biomass requires the responsible enzyme, fungal hyphae or bacterial membranes that excrete hydrolytic enzymes that must first adsorb onto the cellulose surface and then to catalyse the appropriate reactions^3^,^4^,^5^. In both bioactive papers and in enzymatic hydrolysis, the interactions between the cellulose surface and a bio-macromolecule play a critical role in determining the response rate and hence the utility of the process.

Despite the importance of the interactions between bio-macromolecules and cellulose surfaces, there is little useful information available concerning the structure, conformation and density of absorbed bio-macromolecules at cellulose surfaces. The scarcity of information stems primarily from two factors. Firstly, paper and wood both have complex three-dimensional hierarchical structures^6^ and their variable characteristics such as porosity, thickness, and chemical composition render the analysis of adsorption/desorption of antibodies and enzymes extremely difficult. Secondly, the overall chemical compositions and electron/neutron densities of cellulose and most bio-macromolecules are sufficiently close to limit opportunities for the contrast between a cellulose surface and an adsorbed biomolecule when examined by X-ray/neutron reflectometry^7^, spectroscopy and microscopy. These limitations have led various workers to use model cellulose films as a platform to investigate interfacial characteristics^8^,^9^,^10^,^11^,^12^,^13^.

To date, cellulose model films have been prepared from either nanocellulose, or cellulose derivatives using two main approaches: Langmuir-Blodgett (LB) deposition^14^ and spin coating^15^,^16^. The relative performances of these techniques have been reviewed by Kontturi^11^. Native or functionalized films of regenerated and nanofibrillar cellulose have been used to study the adsorption and chemical conjugation of antibodies, and the dynamic adsorption and other features of the adsorbed antibody have been characterized by quartz crystal microbalance (QCM) with dissipation, atomic force microscopy (AFM) and surface plasmon resonance (SPR)^8^,^17^. Factors such as cationicity, ionic strength and pH of the solution in which the macromolecule was dissolved were reported to control antibody/enzyme adsorption^8^,^9^,^18^. Erikson *et al*. measured the adsorption kinetics of *H. insolens*, a cellulase, on thin cellulose films in terms of adsorption mass and film thickness using ellipsometry. They reported full enzyme coverage on cellulose forming a monolayer 25 Å thick adsorbed at Γ_max_ = 5.5 mg/m^2^; the dry cellulose films were 300 Å thick with a roughness of 50 Å and swelled to 820 Å once hydrated^16^.

Despite these advances, our current knowledge of the conformations adopted by biomacromolecules at the cellulose/water interface and of the properties of the adsorbed layers remains limited. Little is known about the interfacial configuration adopted by biomolecules such as enzymes and antibodies once they adsorb onto a cellulose surface, nor on the effect of biomolecule interfacial configuration on functionality. There have been many published examples of the conformation of adsorbed biomacromolecule at the interfaces strongly influencing properties such as reaction rate, selectivity and longevity^19^,^20^,^21^.

While SPR and QCM offer quantification of the adsorption kinetics in real time, atomic force microscopy (AFM), ellipsometry^16^ and reflectometry (X-ray and neutron) have emerged as powerful techniques to probe the morphology of biomolecules at liquid-solid interfaces. Of those techniques, neutron reflectometry (NR) is especially attractive because of its sensitivity to isotopic contrast, specifically between the number density of deuterium (^2^H) and the light isotope of hydrogen (^1^H), between either the biomolecule or the substrate. Under the best conditions, NR can reveal specific important details concerning the adsorbed biomolecule morphology. NR does however suffer from two major drawbacks due to the poor information content of the measurement since it is essentially a ‘one-dimensional technique’, only probing layer thickness composition and uniformity at the Å to μm level. Partial or complete deuteration of either the surface or the biomolecule enhances both level of contrast and the information content of the measurement. Deuteration of cellulose may be achieved more simply and cheaply than deuteration of most bio-macromolecules^22^. The deuterated cellulose can then be processed into smooth, thin films. These films would be an enabling step to exploit the full ability of NR to visualize morphology and quantify the adsorption of many bio-macromolecules on cellulose substrates.

It has been the objective of this study to prepare smooth deuterated cellulose (DC) films from deuterated bacterial cellulose (DBC) suitable for NR studies. This objective has been achieved by growing cultures of *Gluconacetobacter xylinus* in media containing a deuterated carbon source and D_2_O as a solvent. There are no cellulose solvents having sufficient volatility suitable for spin coating and so a number of more soluble chemical derivatives of the deuterated cellulose were prepared and these were dissolved in volatile solvents suitable for spin-coating. Derivatization of deuterated bacterial cellulose into trimethylsilylyl cellulose (TMSC) and cellulose tri-acetate was investigated. Different regeneration methods are available for the different synthesis processes of cellulose film^5^,^10^. In our case, the cellulose films for reflectivity were regenerated from the hydrolysis of TMSC cellulose derivative films under mild conditions as reported by Kent *et al*.^5^. The enhanced contrast was explored by using NR to investigate the structure of a model enzyme, horseradish peroxidase (HRP), absorbed from solution at the deuterated cellulose-water interface.

## Preparation of DC Films from DBC

**Figure 1:**
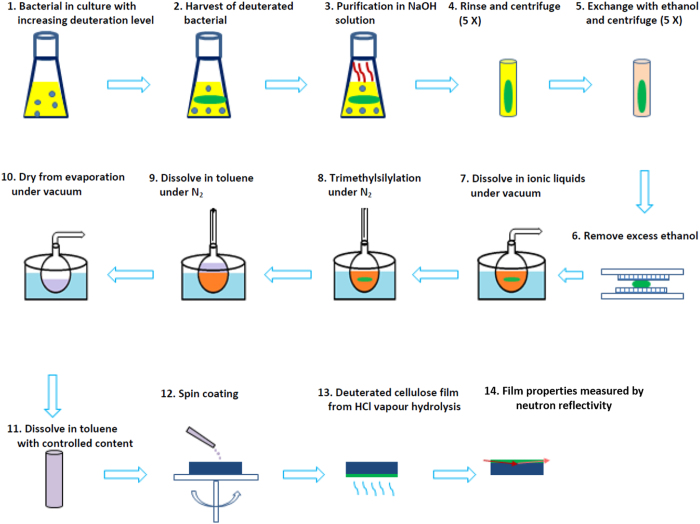
Schematic representation of the procedure used to prepare thin smooth regenerated deuterated cellulose (DC) films from deuterated bacterial cellulose (DBC).

The procedure used to prepare deuterated cellulose films is represented schematically in Fig. 1. It is a three-step process: the first step consists of derivatizing deuterated bacterial cellulose so that it becomes soluble in a volatile organic solvent; the second step is to spin coat a thin, smooth and uniform DBC derivative film onto polished silicon block and the third step involves regenerating the DBC derivative film into a film of deuterated cellulose.

### Deuterated bacterial cellulose growth and characterisation

Deuterated bacterial cellulose pellicles were produced from the *Gluconacetobacter xylinus* strain ATCC 53524 grown on media containing deuterated glycerol as a carbon source dissolved in D_2_O as a solvent. Strains of *Gluconacetobacter xylinus* are prolific in bacterial cellulose production, forming a pad or a membrane of cellulose called a pellicle on the surface of the culture medium. Unlike most plant-derived cellulose, the bacterial cellulose is not biosynthesized as a composite with other cell wall polymers (lignin, hemicelluloses), but is extruded into the growth medium from where it can be isolated and purified readily^23^.

**Figure 2:**
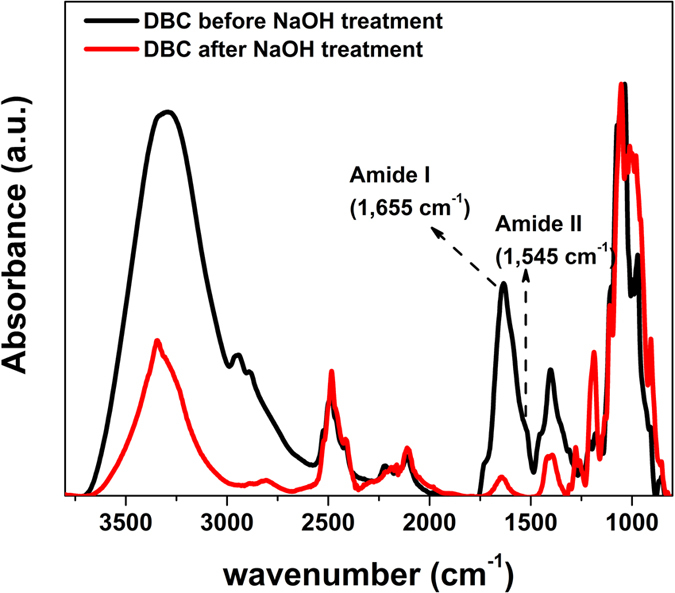
ATR-FTIR spectra of dried deuterated bacterial cellulose before and after NaOH treatment to remove protein and other cellular material. The spectra are normalized to the maximum peak height for clarity of presentation.

The details and characterisation of bacterial cellulose pellicles using different carbon sources containing hydrogen atoms in natural isotopic abundances have been were published elsewhere^24^. Bacteria were initially cultivated in Hestrin and Schramm media with glycerol as the carbon source. The media consisted of 20 g/L carbon source, 5 g/L peptone, 5 g/L yeast extract, 2.7 g/L NaHPO_4_ and 1.15 g/L of citric acid in H_2_O initially for adaptation of the bacteria and then moved to the deuterated medium through several increments of increasing deuterated glycerol and D_2_O in the solvent. The pH of the medium was adjusted to 5.0 at the start of growth. Cellulose pellicles were cultivated with increasing amounts of deuteration before final harvesting. This procedure allows in principle production of cellulose films with multiple levels of deuteration. The final DBC pellicles were first rinsed with distilled water to remove excess medium, and then heated (90 °C) in 0.1 M NaOH solution for 30 min to inactivate attached bacterial cells and remove proteins. After boiling, the pellicles were purified by extensive washing in distilled water at room temperature and centrifuged (5000 rpm, 10 min) several times until the pH of the water reached 7.0. Small samples of DBC were collected before and after boiling and subjected to vacuum drying for two days for attenuated total reflectance Fourier transforms infrared spectroscopy (ATR-FTIR). Figure 2 shows the ATR-FTIR spectra of the DBC before and after treatment with NaOH solution. Absence of two signature bands of attributable to protein (amide I at 1,655 cm^−1^ and amide II at 1,545 cm^−1^) after the treatment with NaOH, washing and freeze drying^25^ confirmed the efficiency of removal of bacterial cells and debris from the pellicles.

Oven-dried weights of samples were determined after drying overnight at 105 °C. The remaining wet DBC samples were repeatedly solvent exchanged with 100% ethanol by placing the wet suspension in a 50 mL tube for 10 min with 100% ethanol (40 mL), and then centrifuging (5000 rpm, 10 min). Removal of the aqueous ethanol supernatant and repetition of the process five times gave a suspension of DBC in ethanol that was essentially anhydrous. Finally, the excess ethanol was removed from the DBC by pressing against a nylon fabric mesh on top of a blotting paper. This DBC sample was kept at 4 °C in a closed container and used without further drying for preparing cellulose acetates and trimethylsilylation (TMS) of DBC that could be dissolved in volatile solvents.

**Figure 3:**
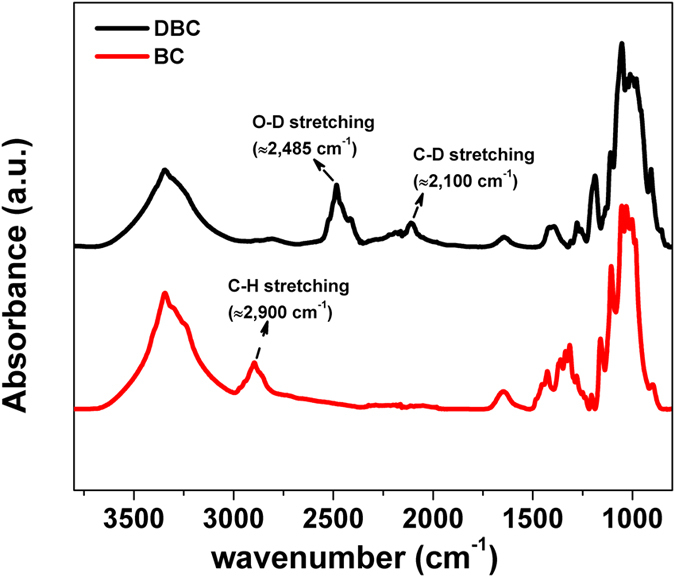
ATR-FTIR spectra of deuterated bacterial cellulose (top Black) and natural bacterial cellulose (bottom red). Each spectrum was normalized to maximum peak height and offset for clarity.

The ATR-FTIR spectrum of deuterated (DBC) and natural bacterial cellulose (BC) as a reference were compared in Fig. 3 and assignments are given in Table 1. Those assignments agree to those in the literature^26^. Infra-red bands attributed to OH-stretching are located at approximately 3,500 cm^−1^ and 3,300 cm^−1^ in the samples. The majority of the bands corresponding to the CH-stretching modes are located between 3,000 cm^−1^ and 2,800 cm^−1^, and the expected absorption bands were in fact observed in the BC spectrum (Fig. 3). These bands were largely absent in the corresponding spectra of the DBC sample consistent with the expected non-lability of the C-D bond to undergo exchange. Bands around 2,485 cm^−1^ and 2,100 cm^−1^, corresponding to O-D stretching and C-D stretching respectively and consistent with the expected isotopic shift for IR bands, were recorded in the DBC sample (Fig. 3) indicating that only a small proportion of the deuterium attached to oxygen had been exchanged for hydrogen during contact with water and ethanol. These data provide strong evidence that a major proportion of deuterium was incorporated into the DBC by the bacteria and that O-D to O-H exchange in heavy water at pH 7 is relatively slow.

### Deuterated Cellulose films from DBC

DBC as purified above was used for preparing solution of the (deuterated) cellulose derivative, trimethylsilylyl cellulose (TMSC), for spin coating and subsequent deuterated cellulose film preparation. This process-required trimethylsilylation of cellulose^28^,^29^, and achieved as follows: Ionic liquid BMIM chloride (10 g) was dried by placing it in a 25 mL single-necked round-bottomed flask and heated at 120 °C under vacuum (500 Pa) for 16 h. In order to minimise ingress of moisture, the flask was allowed to cool to room temperature and then fitted with a condenser, magnetic stir bar and silicone oil bubbler that enabled the BMIM chloride to be kept under an atmosphere of dry high purity nitrogen. Hexamethyl disilazane (2 mL) was added to the BMIM chloride and the mixture stirred overnight at 100 °C. The condenser and bubbler were removed, and then volatiles were removed by rotary evaporation under vacuum at 170 °C (3 h, 500 Pa).

A 25 mL three-necked, round-bottomed flask equipped with a reflux condenser and a magnetic stir bar was charged with DBC (0.1 g, 0.61 mmol equivalents) and BMIM chloride (2.5 g dried as above). The mixture was stirred at 100 °C under vacuum (500 Pa). A clear solution of deuterated TMSC in the ionic liquid was obtained after 6 h. The flask was then equipped with a silicone bubbler and an inlet for dry nitrogen and was flushed with dry nitrogen for 30 min. Following dissolution of the cellulose, hexamethyl disilazane (1.8 g) was added and the mixture stirred at 100 °C for 2 h. Following this period, anhydrous toluene (8 mL) was added and the mixture stirred at 100 °C for a further 16 h. At that point stirring was stopped and the solution allowed cooling to room temperature. The toluene phase was separated from the ionic liquid using a syringe and the ionic liquid was extracted twice with anhydrous toluene (2 × 8 mL). The combined toluene fractions were reduced to a volume of 4 mL by rotary evaporation under vacuum (500 Pa). The product was precipitated by pouring the toluene solution into anhydrous methanol (50 mL). The product was isolated by filtration, washed three times with anhydrous methanol (3 × 5 mL) and dried at 60 °C, 5 Pa to give trimethylsilyl deuterated bacterial cellulose as a white amorphous solid (0.15 g). The trimethylsilylyl cellulose (TMSC 100 mg), was dissolved in toluene (10 mL) and the solution was placed on a cleaned silicon block and then spin-coated at 4000 rpm for 30 sec. The deuterated TMSC films were hydrolysed to deuterated cellulose films by exposure to vapours above 1M aqueous HCl in a closed container at 20 °C for 15 mins^30^.

### Characterization of deuterated cellulose films

**Figure 4:**
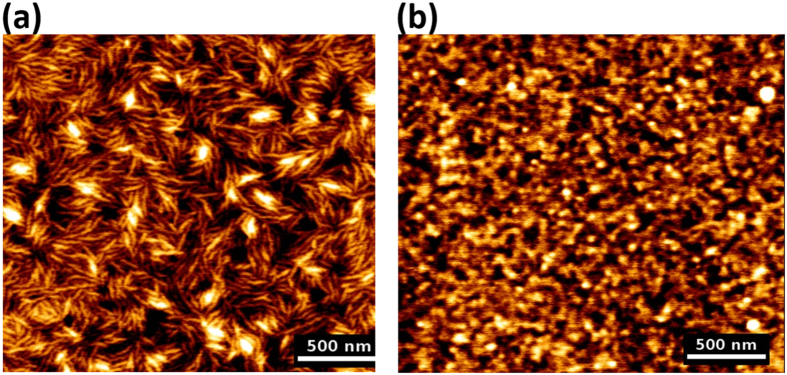
Atomic force microscope (AFM) images of spin coated deuterated bacterial cellulose films (**a**) Before regeneration (**b**) After regeneration in vapour above 1M aqueous HCl.

The quality of the DC films prepared by spin coating as described in the Experimental section was first characterised by Atomic Force Microscopy (AFM). The smoothness of the film before and after regeneration with acid vapours (Fig. 4) was characterised. Both AFM images show uniform and smooth films with a root mean square (RMS) roughness of 17 Å and 11 Å before (Fig. 4a) and after (Fig. 4b) regeneration, respectively.

**Figure 5:**
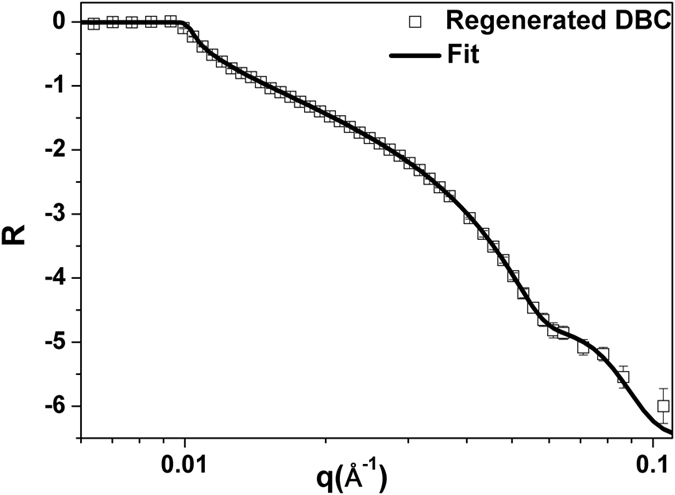
Neutron reflectivity from a regenerated deuterated bacterial cellulose film at the air/solid interface. The solid line shows model NR from a single cellulose layer model.

Specular neutron reflection (NR) is a very useful technique for direct investigation of phenomena at the solid/water and solid/air interfaces^5^,^31^,^31^. The technique can also be employed to monitor the interfacial interactions between cellulose and bio-macromolecules under controlled conditions. Figure 5 shows the NR measurements on the DC film at the air/film interface. The fringes observed in the NR measurements related to the SLD difference, thickness and smoothness of film. For further analysis, the NR curves were fitted using IgorPro based the MOTOFIT macro^33^. The model consists of a number of slabs of known scattering length density, thickness and interfacial roughness. The front and back of the model are effectively infinite and depending on the measurement geometry, on one side Si, and on the other side either air or D_2_O. A further modelling parameter may be introduced, in the solvent penetration model, where the slab contains a homogenous mixture of solvent and polymer. In this case the SLD of the slab is determined by the volume fraction of water, since the SLD of this slab will be a linear combination of the solvent and cellulose SLD’s^32^,^33^,^34^,^35^.

In all cases, modelling the NR curve was underpinned by a pre-existing SiO_2_ layer on the bare Si block. The resulting fitted parameters for subsequent samples (layer thickness, interfacial roughness and SLD) are shown in Table 2. The thickness of the deposited DC film measured in air is 90 Å with a roughness of 15 Å. The resulting SLD of the DBC film is 5.5 × 10^−6 ^Å^−2^ which is similar to the theoretically calculated SLD of 5.58 × 10^−6 ^Å^−2^ for deuterated cellulose having a chemical composition of C_6_H_3_D_7_O_5_ and density of 1.5 g.cm^−3^.

## Visualisation of Bio-Molecules

**Figure 6:**
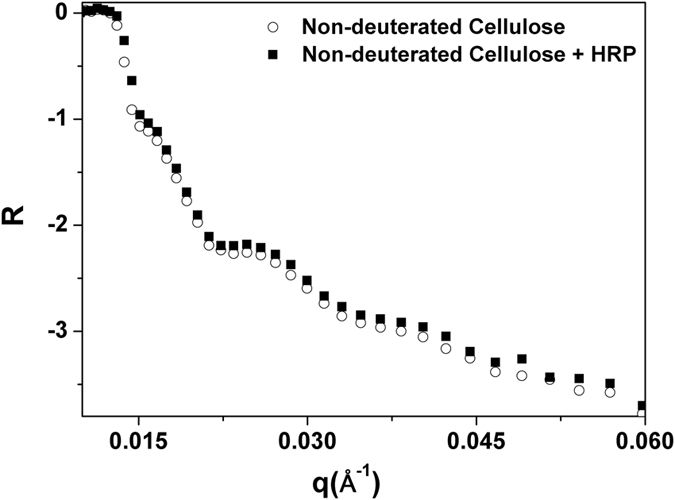
Regenerated cellulose (non-deuterated) with and without enzyme (HRP) measured in D_2_O buffer. The open circle shows the cellulose film and the filled squares shows the same with a layer of HRP adsorbed on it.

Adsorption and visualisation of bio-macromolecules on deuterated and natural isotopic abundance (non-deuterated) cellulose was studied by neutron reflectivity. Horseradish peroxidase (HRP) was used as a model bio-macromolecule to investigate the utility of NR in revealing adsorption conformation information for biomolecules adsorbed at the solid solution interface. HRP is an alpha-helical enzyme containing a heme group. It is considered a suitable model system because of its well-known structure, mechanistic understanding^36^, relatively stability and high potential for biotechnological use^37^. The limitations in applying NR to study protein adsorption on smooth natural isotopic abundance cellulose films were illustrated by adsorbing HRP onto a thin smooth cellulose film prepared by spin coating commercial cellulose triacetate onto a silicon wafer and then hydrolysing the acetate using the procedure described by Su *et al*.^20^. Figure 6 shows the NR curves for the resulting regenerated non deuterated cellulose film with and without HRP measured in D_2_O buffer. The number of fringes in the NR curve for natural isotopic abundance cellulose film (open circle) clearly shows that the films are smooth and thick. The curve indicated with filled squares shows the NR profile for the same film with HRP adsorbed on its surface. There is no observable difference in NR curves with and without HRP adsorption; both curves are indistinguishable. This is due to the lack of contrast between the adsorbed layer of HRP and the cellulose layer, possibly compounded by the expected very low film thickness of the adsorbed HRP layer. It was therefore necessary to use deuterated cellulose to gain contrast and clear visualization of biomolecule adsorption conformation.

### Adsorption of Horseradish Peroxidase (HRP) on DC film

**Figure 7:**
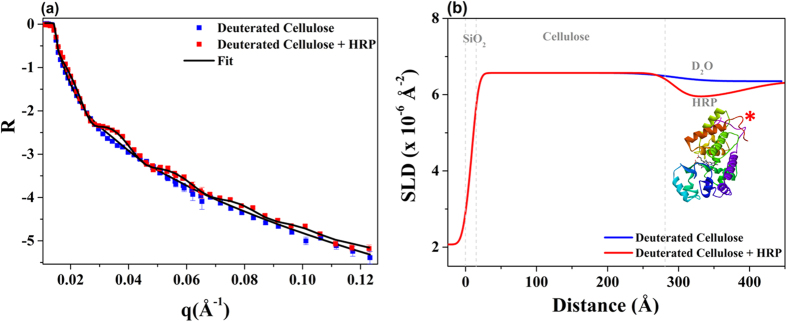
Reflectivity data from the regenerated deuterated cellulose film without (blue square) and with adsorbed HRP (red square) over a D_2_O sub-phase (**a**). The solid line shows the fit of the NR data. (**b**) Model SLD profiles of the models used for fitting the respective NR data for DC film (blue partially overlaid with red) and DC film with adsorbed HRP (red). The structure of HRP (*) was taken from the article published by Carlsson *et al*.^38^.

In order to insure full or maximum surface coverage of HRP at the liquid-cellulose interface, HRP was adsorbed from solution and equilibrated onto DC film under conditions of excess HRP. Figure 7(a) shows the NR curves for DC films with and without a sorbed HRP layer over D_2_O as a sub-phase. Comparing the NR curve of the DC film after HRP adsorption (red squares) to the original DC film (blue squares) – shows the appearance of pronounced fringes from the film formed by absorbed HRP (red squares). These fringes are attributable to the appearance of a layer with noticeable SLD difference from the DC film. For further analysis, the NR curve of the DC film at the D_2_O/film interface was fitted with a single layer model. An additional layer was included to fit the NR curve with the HRP adsorbed onto the DC film surface. Figure 7(a) shows the fitted NR curves and Fig. 7(b) represents the model of SLD profile of DC film and DC/HRP used for NR curve fitting. The SLD plot clearly reveals the contrast difference between the DC cellulose layer and the layer containing the HRP enzymes and allows clear visualisation.

Table 2 regroups the parameters obtained from fitting the NR curves. SLD of the non-deuterated cellulose (C_6_H_10_O_5_) is typically about 1.7 × 10^−6 ^Å^−2^. Here, for DC film in the presence of D_2_O, the resulting SLD is about 7.1 ×10^−6 ^Å^−2^ which corresponds to the composition lying between C_6_HD_9_O_5_ and C_6_D_10_O_5_. These data provide strong evidence that the regenerated deuterated cellulose films in contact with the buffered D_2_O are highly deuterated (between 90–100%). Moreover, the thickness of DC film increased from 98 ± 10 Å in air to 280 ± 15 Å upon swelling in buffered D_2_O; this corresponds to a swelling factor of about three showing that cellulose at the liquid interface becomes a hydrogel.

Results from HRP adsorption onto the DC film reveals an HRP layer thickness of about 98 ± 10 Å with a SLD of 3.9 × 10^−6 ^Å^−2^. The measurements were performed in the presence of D_2_O and the results show an adsorbed volume fraction for HRP of about 20%. This suggests either that HRP does not adsorb as a full monolayer, or that the molecules are strongly hydrated and adopt an open conformation when adsorbed into cellulose. Given that the molecular function of HRP continues when the molecule is adsorbed on a cellulose substrate^39^,^40^ it is unlikely that the protein is unfolded, since this function is heavily dependent on the correct folding.

## Discussion

### Preparation and characterization of deuterated smooth cellulose films

The deuterated cellulose films preparation methodology developed here was an adaptation of the methods of Greber and Paschinger^41^, Cooper^42^ and Cheng *et al*.^5^ Earlier, cellulose thin films using the methodology reported by Eriksson *et al*. and relying on the direct cellulose dissolution in DMAc/LiCl were also tested^16^; however for (deuterated) bacterial cellulose it produced films of relatively high roughness as compared to the methodology selected. The DBC obtained from Gluconacetobacter xylinus strain ATCC 53524, using deuterated glycerol and D_2_O as the solvent for the media was fully dissolved into an ionic fluid, and then reacted to completion (degree of substitution (DS = 3) into trimethylsilyl cellulose. This derivative was readily soluble in toluene, which is an excellent solvent for spin coating. ATR-FTIR investigations (Fig. 3) reveals the large proportion of deuterium atoms have replaced the H atoms. NR investigations (Fig. 7(a) and Table 2) shows that the DC films over a D_2_O sub-phase have a high deuteration level close to 100%. Our study to optimize deuterated cellulose films preparation for NR application led to the observations that the bacterial cellulose is more difficult to dissolve than cellulose from woody lignocellulose origin; this is probably due to a higher molecular weight and a crystallite morphology^43^.

### Variation of cellulose contrast for biomolecule visualization

**Figure 8:**
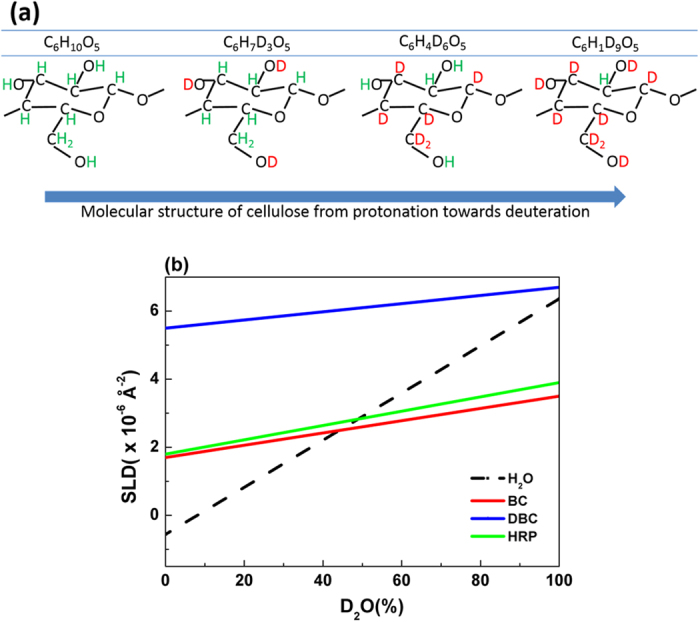
(**a**) Molecular structure of cellulose at different deuteration levels. (**b**) Scattering length densities (SLDs) from HRP, cellulose and deuterated cellulose as a function of D_2_O content in the D_2_O/H_2_O mixture. The labile protons from the 3 hydroxy groups per glucose are exchanged reflecting the D_2_O/H_2_O ratio of the solvent. The crossing point of the water line with the protein and cellulose represents the “matching” point. The SLD for HRP was calculated from the fitting of the NR curve.

Deuteration, achieved either by manipulating the contrast of water in sub-phase, or by manipulating the biosynthesis of the macromolecule, is useful to reveal the biomolecule adsorption conformation at the solid-liquid interface by using NR. When natural isotopic abundance water (H_2_O) is replaced by heavy water (D_2_O), all of the hydroxy groups may exchange so that isotopic mixture reflects that of the subphase. Figure 8a shows the deuteration arrangement on each anydroglucose unit for different combinations of solvent/sub-phase composition and levels of cellulose biodeuteration. The hydrogens bound to a carbon atom cannot exchange, but those linked to a nitrogen or oxygen atom can if they are accessible to the solvent^44^,^45^. In these films, we have shown that all hydroxyl groups are available for exchange. In proteins, generally it is assumed that about 10% of all exchangeable groups are available on the exterior of the protein^46^, this is consistent with our observation of a clear layer with a SLD approaching that calculated from the crystal structure (PDB file 1H5A^36^) and the program CRYSON^46^.

Changing the H_2_O/D_2_O ratio of the liquid phase is a simple way to control the level of deuteration; the neutron SLD range offered is however restricted. Cellulose bio-deuteration offers a very attractive option for achieving a wider range of SLD contrast and measuring physical properties of any adsorbed materials, as demonstrated above. The important factor is that while only oxygen-, nitrogen- and sulfur-bound hydrogen atoms in HRP will exchange in D_2_O, or mixed H_2_O/D_2_O solutions, relatively facile bacterial biosynthesis of cellulose in which the carbon-bound hydrogen is replaced with deuterium provides a route to considerably higher SLD values in the underlying cellulose films. Additionally, it is entirely possible that spectroscopic analysis of fully and partially deuterated cellulose, in either film or fibrous form, associated with adsorbed enzymes and antibodies using advanced two-dimensional nuclear magnetic resonance (NMR) methods may yield further insights into the types of interactions and molecular conformations involved. Bacterial biosynthesis of fully deuterated cellulose clearly opens up a many avenues for studying how cellulose interacts with biologically important molecules.

The SLD of natural isotopic abundance cellulose (C_6_H_10_O_5_) is 1.7 × 10^−6 ^Å^−2^ and after exchange of the three labile hydroxy protons per anhydroglucose unit, the SLD 3.4 × 10^−6 ^Å^−2^ (C_6_H_7_D_3_O_5_) in D_2_O. The SLD of fully bio-deuterated cellulose (C_6_D_10_O_5_) in equilibrium with D_2_O is 7.1 × 10^−6 ^Å^−2^. Figure 8b shows the SLD of HRP, natural isotopic abundance cellulose and deuterated cellulose as a function of D_2_O content. The labile protons on the hydroxy groups reflect the D_2_O content of the solvent. The average value of SLD for HRP was calculated based on the number density of atoms and 10% exchange using the program CRYSON^46^. Over the range of D_2_O content, there is only a small difference in SLD value between cellulose having varying H/D ratios (red line) and the corresponding ratios in HRP (green line). This hinders visualization of proteins adsorbed onto natural isotopic abundance cellulose surface. On increasing the deuteration level of cellulose (blue line), the SLD difference increases significantly and high contrast levels for proteins with respect to deuterated cellulose can be achieved. This contrast enables the precise measurement of the thickness of a very thin layer of proteins adsorbed on cellulose, such as the enzymes and antibodies used in paper diagnostics.

The thickness of the adsorbed HRP layer at the liquid-cellulose interface measured by NR was 98 ± 10 Å with a volume fraction of 20% of the protein. Various representations of the three-dimensional structure of a single molecule are reported: for HRP in neutral buffer solution determined by *in-situ* ECSTM the dimensions of surface adsorbed particle was 68 × 44 × 40 Å^3 ^47; according to X-ray crystallography^36^ the dimensions of the molecule pack in a crystal are 40 × 68 × 117 Å^3 ^48. Although there is considerable discrepancy between the dimensions of HRP molecules adsorbed on a surface and that packed in a crystal, the thickness of the layer measured accommodates even the extreme dimensions of the protein. HRP molecules adsorbed on a surface might form structures that are random, or fully or partially ordered if the dipolar and H-bonding interactions are sufficiently strong. Clearly, whether the adsorption takes place under kinetic, or thermodynamic control may have a strong influence on the degree of order in the protein layer. This interesting aspect of protein adsorption will be further studied by our group. In the present case, with the partial surface coverage achieved, surface relaxation of HRP is expected with some agglomeration. The low HRP surface coverage was anticipated from the low HRP affinity for cellulose, driven by non-electrostatic (van der Waals) forces. Agglomeration of HRP molecules may form irregular structures surrounded with a thick layer of D_2_O, which results in 80% of solvent fraction in the film. Our experiments (Fig. 6) confirm that cellulose deuteration using bacterial biosynthesis is a key method for visualizing and quantifying thin layers of enzyme adsorbed onto cellulose surfaces.

## Conclusion

In the present study, thin, smooth deuterated cellulose (DC) films have been prepared and these provide sufficient contrast using neutron reflectometry to visualize and quantify absorbed layers of horseradish peroxidase (HRP) as an example of an important class of bio-macromolecules. First, deuterated bacterial cellulose (DBC) was obtained by growing Gluconacetobacter xylinus strain ATCC 53524 in deuterated glycerol in D_2_O as a carbon source. Afterwards, two strategies were compared to synthesize cellulose derivatives which can dissolve in organic solvents (acetone and toluene) for spin coating: either acetylation or trimethylsilylation in ionic liquid (1-Butyl-3-methylimidazolium chloride). The trimethylsilylation of deuterated bacterial cellulose allows high yield of soluble product, and was used to prepare toluene solution for spin-coating onto silicon substrate. The resultant thin film was hydrolysed into deuterated cellulose in HCl vapour. Thin (100–200 Å) and smooth (RMS = 10–20 Å) cellulose films resulted. Adsorption of an enzyme, horseradish peroxidase (HRP) onto the surface of DC film surface could clearly be visualized and quantified by neutron reflectivity analysis. Data analysis showed the thickness of adsorbed HRP layer at the liquid-cellulose interface is 98 ± 10 Å, with roughness of 17 ± 5 Å and with a volume fraction of 20%; this corresponds to partial surface coverage of HRP aggregates. The method developed enables preparation of thin cellulose films with controllable deuteration levels. Control over the level of deuteration can provide a wide range of controllable scattering contrasts that greatly enhances the investigation of the interactions between cellulose and biologically important bio-macromolecules.

## Experimental

### Materials

1-Butyl-3-methylimidazolium chloride (BMIM chloride, ≥98.0%, HPLC), hexamethyldisilazane (HMDS, ReagentPlus, 99.9%), toluene (anhydrous, 99.8%), acetone (AR, ≥99.5%), cellulose acetate (M_r_~29,000, acetyl groups substitution ~40%) were all purchased from Sigma-Aldrich. Polished silicon wafers (50.8 mm diameter, 12 mm and 0.5 mm height, n-type Si:P, [100]) were purchased from El-Cat Inc. The wafers were cleaned by soaking in a mixture of ammonium hydroxide, hydrogen peroxide and water (volume ratio = 1:1:5) at 70 °C for 15 min, allowed to air dry in a dust-free environment and used as substrates for spin coating. A spin coater from Laurell Technologies Co., Pennsylvania, model WS-650HZB-23NPPB-UD-3 was used.

### Preparation of thin smooth cellulose films from cellulose acetate

Non-deuterated cellulose films were prepared by spin coating solutions of commercial cellulose acetate in acetone onto silicon wafers and then subjecting the films to alkaline hydrolysis as described by Su *et al*.^20^.

### Physical Measurements

Infrared spectra (IR) were obtained by the Fourier Transform Method in attenuated total reflectance geometry (ATR-FTIR, Perkin-Elmer, Spectrum One with an STI “Thunderdome” ATR system). The spectra were the result of the accumulation of 64 scans, at a resolution of 4 cm^−1^ in the range from 4,000 to 650 cm^−1^.

AFM measurements were made using a JPK Nanowizard 3 in AC (intermittent contact) mode. The piezoelectric scanner elements were monitored by capacitive sensors to ensure accurate reporting of height, z, and x-y lateral distances. Cantilevers used were Bruker NCHV model tapping mode levers, with nominal resonant frequencies of 340 kHz and spring constants of 20–80 N/m. Imaging was performed with a force set-point of <1 nN. In post-acquisition processing, images were ‘flattened’ to arrange the scan lines in a plane only by the subtraction of a linear equation from each scan line in the JPK image analysis software. Height images converted the lateral (z) scale acquired by the AFM z-piezoelectric drive capacitive feedback loop to a colour for ease of presentation.

Neutron reflectometry (NR) measurements were performed at the PLATYPUS beamline of the Australian Nuclear Science and Technology Organization (ANSTO, Sydney, Australia)^33^. Measurements were performed at three different angles (0.5, 0.85 and 3.8 degrees). Data reduction and modelling of the NR curves were conducted using the IgorPro based macro MOTOFIT^33^.

## Additional Information

**Publisher's note**: Springer Nature remains neutral with regard to jurisdictional claims in published maps and institutional affiliations.

**How to cite this article**: Su, J. *et al*. Smooth deuterated cellulose films for the visualisation of adsorbed bio-macromolecules. *Sci. Rep*. **6**, 36119; doi: 10.1038/srep36119 (2016).

## Figures and Tables

**Table 1:** Characterization of ATR-FTIR spectra for deuterated and natural bacterial cellulose.

Band assignment ⇒⇓ Wavenumber (cm^−1^)	O-H stretching	C-H stretching	C-H bending deformation	O-H In plane bending	C-O stretching	C-O-C vibration	C-D Stretching	O-D stretching
Natural Bacterial Cellulose^26^,^27^	3200–3480	2900	1454, 1361, 1315, 1277	1208	1054	1108	—	—
Deuterated Bacterial Cellulose	3337	2888 (weak)	1420, 1308, 1277 (weak)	1186	1054	1110	2100	2485

**Table 2:** Surface characterization parameters resulting from fitting the NR curves for the DC film measured in air, D_2_O and with the adsorbed HRP layer.

Sample	Thickness (Å)	SLD (10^−6 ^Å^−2^)	Volume fraction	Roughness (Å)
DC/Air	90	5.5	NA	15
DC/D_2_O	280	7.1	33 (cellulose)	8
HRP	98	3.9	20 (HRP)	17

## References

[b1] Ngo Y. H., Li D., Simon G. P. & Garnier G. Paper surfaces functionalized by nanoparticles. Advances in Colloid and Interface Science 163, 23–38 (2011). 21324427 10.1016/j.cis.2011.01.004

[b2] Then Whui L. & Garnier G. Paper diagnostics in biomedicine. In: Reviews in Analytical Chemistry (ed^(eds) (2013).

[b3] Brady S. K., Sreelatha S., Feng Y., Chundawat S. P. S. & Lang M. J. Cellobiohydrolase 1 from Trichoderma reesei degrades cellulose in single cellobiose steps. Nature Communications 6, (2015). 10.1038/ncomms10149 PMC4682103 26657780

[b4] Cheng G. . Interactions of Endoglucanases with Amorphous Cellulose Films Resolved by Neutron Reflectometry and Quartz Crystal Microbalance with Dissipation Monitoring. Langmuir 28, 8348–8358 (2012). 22554348 10.1021/la300955q

[b5] Cheng G. . Neutron Reflectometry and QCM-D Study of the Interaction of Cellulases with Films of Amorphous Cellulose. Biomacromolecules 12, 2216–2224 (2011). 21553874 10.1021/bm200305u

[b6] Gibson L. J. The hierarchical structure and mechanics of plant materials. J R Soc Interface 9, 2749–2766 (2012). 10.1098/rsif.2012.0341 PMC3479918 22874093

[b7] Su J. . Adsorption of cationic polyacrylamide at the cellulose-liquid interface: A neutron reflectometry study. Journal of Colloid and Interface Science 448, 88–99 (2015). 25723785 10.1016/j.jcis.2015.02.008

[b8] Orelma H., Teerinen T., Johansson L. S., Holappa S. & Laine J. CMC-modified cellulose biointerface for antibody conjugation. Biomacromolecules 13, 1051–1058 (2012). 22360491 10.1021/bm201771m

[b9] Orelma H., Johansson L. S., Filpponen I., Rojas O. J. & Laine J. Generic Method for Attaching Biomolecules via Avidin-Biotin Complexes Immobilized on Films of Regenerated and Nanofibrillar Cellulose.Biomacromolecules 13, 2802–2810 (2012). 22831169 10.1021/bm300781k

[b10] Aulin C. . Nanoscale Cellulose Films with Different Crystallinities and Mesostructures-Their Surface Properties and Interaction with Water. Langmuir 25, 7675–7685 (2009). 19348478 10.1021/la900323n

[b11] Kontturi E., Tammelin T. & Osterberg M. Cellulose - model films and the fundamental approach. Chem Soc Rev 35, 1287–1304 (2006). 10.1039/b601872f 17225889

[b12] Wagberg L. O. M. & Enarsson L. Interactions at Cellulose Model Surfaces. Encyclopedia of Surface and Colloid Science 1, 1–19 (2010).

[b13] Salas C., Nypelö T., Rodriguez-Abreu C., Carrillo C. & Rojas O. J. Nanocellulose properties and applications in colloids and interfaces. Current Opinion in Colloid & Interface Science 19, 383–396 (2014).

[b14] Rundlof M., Karlsson M., Wagberg L., Poptoshev E., Rutland M. & Claesson P. Application of the JKR method to the measurement of adhesion to Langmuir-Blodgett cellulose surfaces. Journal of Colloid and Interface Science 230, 441–447 (2000). 11017752 10.1006/jcis.2000.7108

[b15] Eriksson M., Notley S. M. & Wagberg L. Cellulose thin films: Degree of cellulose ordering and its influence on adhesion. Biomacromolecules 8, 912–919 (2007). 17319721 10.1021/bm061164w

[b16] Eriksson J., Malmsten M., Tiberg F., Callisen T. H., Damhus T. & Johansen K. S. Model cellulose films exposed to H-insolens glucoside hydrolase family 45 endo-cellulase - the effect of the carbohydrate-binding module. Journal of Colloid and Interface Science 285, 94–99 (2005). 15797401 10.1016/j.jcis.2004.10.042

[b17] Orelma H., Filpponen I., Johansson L. S., Laine J. & Rojas O. J. Modification of Cellulose Films by Adsorption of CMC and Chitosan for Controlled Attachment of Biomolecules. Biomacromolecules 12, 4311–4318 (2011). 22035370 10.1021/bm201236a

[b18] Norde W., Macritchie F., Nowicka G. & Lyklema J. Protein Adsorption at Solid Liquid Interfaces - Reversibility and Conformation Aspects. Journal of Colloid and Interface Science 112, 447–456 (1986).

[b19] Khan M. S. & Garnier G. Direct measurement of alkaline phosphatase kinetics on bioactive paper. Chem Eng Sci 87, 91–99 (2013).

[b20] Khan M. S., Haniffa S. B. M., Slater A. & Garnier G. Effect of polymers on the retention and aging of enzyme on bioactive papers. Colloid Surf B-Biointerfaces 79, 88–96 (2010). 10.1016/j.colsurfb.2010.03.034 20417074

[b21] Khan M. S., Li X., Shen W. & Garnier G. Thermal stability of bioactive enzymatic papers. Colloid Surf B-Biointerfaces 75, 239–246 (2010). 10.1016/j.colsurfb.2009.08.042 19775873

[b22] Russell R. A., Garvey C. J., Darwish T. A., Foster L. J. R. & Holden P. J. Chapter Five - Biopolymer Deuteration for Neutron Scattering and Other Isotope-Sensitive Techniques. In: Methods in Enzymology (ed^(eds Zvi K.). Academic Press (2015). 10.1016/bs.mie.2015.06.015 26577729

[b23] Iguchi M., Yamanaka S. & Budhiono A. Bacterial cellulose—a masterpiece of nature’s arts. Journal of Materials Science 35, 261–270 (2000).

[b24] Mikkelsen D., Flanagan B. M., Dykes G. A. & Gidley M. J. Influence of different carbon sources on bacterial cellulose production by Gluconacetobacter xylinus strain ATCC 53524. J Appl Microbiol 107, 576–583 (2009). 19302295 10.1111/j.1365-2672.2009.04226.x

[b25] Giordano M., Kansiz M., Heraud P., Beardall J., Wood B. & McNaughton D. Fourier transform infrared spectroscopy as a novel tool to investigate changes in intracellular macromolecular pools in the marine microalga Chaetoceros muellerii (Bacillariophyceae). J Phycol 37, 271–279 (2001).

[b26] Marechal Y. & Chanzy H. The hydrogen bond network in I-beta cellulose as observed by infrared spectrometry. Journal of Molecular Structure 523, 183–196 (2000).

[b27] Higgins H. G., Stewart C. M. & Harrington K. J. Infrared spectra of cellulose and related polysaccharides. Journal of Polymer Science 51, 59-& (1961).

[b28] Mormann W. Silylation of cellulose with hexamethyldisilazane in ammonia - activation, catalysis, mechanism, properties. Cellulose 10, 271–281 (2003).

[b29] Mormann W. & Wagner T. Silylation of cellulose and low-molecular-weight carbohydrates with hexamethyldisilazane in liquid ammonia. Macromol Rapid Comm 18, 515–522 (1997).

[b30] Mohan T. . Wettability and surface composition of partly and fully regenerated cellulose thin films from trimethylsilyl cellulose. Journal of Colloid and Interface Science 358, 604–610 (2011). 21458821 10.1016/j.jcis.2011.03.022

[b31] Su J. . Adsorption of cationic polyacrylamide at the cellulose-liquid interface: a neutron reflectometry study. J Colloid Interf Sci (2015). 10.1016/j.jcis.2015.02.008 25723785

[b32] Kontturi E. . Amorphous Characteristics of an Ultrathin Cellulose Film. Biomacromolecules 12, 770–777 (2011). 21294555 10.1021/bm101382q

[b33] Nelson A. Co-refinement of multiple-contrast neutron/X-ray reflectivity data using MOTOFIT. J Appl Crystallogr 39, 273–276 (2006).

[b34] Cowsill B. J. . Interfacial Structure of Immobilized Antibodies and Perdeuterated HSA in Model Pregnancy Tests Measured with Neutron Reflectivity. Langmuir 30, 5880–5887 (2014). 24788076 10.1021/la4036166

[b35] Fragneto-Cusani G. Neutron reflectivity at the solid/liquid interface: examples of applications in biophysics. J Phys-Condens Mat 13, 4973–4989 (2001).

[b36] Berglund G. I., Carlsson G. H., Smith A. T., Szoke H., Henriksen A. & Hajdu J. The catalytic pathway of horseradish peroxidase at high resolution. Nature 417, 463–468 (2002). 12024218 10.1038/417463a

[b37] Veitch N. C. Horseradish peroxidase: a modern view of a classic enzyme. Phytochemistry 65, 249–259 (2004). 14751298 10.1016/j.phytochem.2003.10.022

[b38] Carlsson G. H., Nicholls P., Svistunenko D., Berglund G. I. & Hajdu J. Complexes of horseradish peroxidase with formate, acetate, and carbon monoxide. Biochemistry-Us 44, 635–642 (2005). 10.1021/bi0483211 15641789

[b39] Roda A., Guardigli M., Russo C., Pasini P. & Baraldini M. Protein microdeposition using a conventional ink-jet printer. Biotechniques 28, 492–496 (2000). 10723562 10.2144/00283st07

[b40] Di Risio S. & Yan N. Adsorption and inactivation behavior of horseradish peroxidase on cellulosic fiber surfaces. Journal of Colloid and Interface Science 338, 410–419 (2009). 19643429 10.1016/j.jcis.2009.07.005

[b41] Greber G. & Paschinger O. Silyle Derivates of Cellulose. Papier 35, 547–554 (1981).

[b42] Cooper G. K., Sandberg K. R. & Hinck J. F. Trimethylsilyl Cellulose as Precursor to Regenerated Cellulose Fiber. J Appl Polym Sci 26, 3827–3836 (1981).

[b43] Shibazaki H., Kuga S., Onabe F. & Usuda M. Bacterial cellulose membrane as separation medium. J Appl Polym Sci 50, 965–969 (1993).

[b44] Campbell S., Rodgers M. T., Marzluff E. M. & Beauchamp J. L. Deuterium exchange reactions as a probe of biomolecule structure. Fundamental studies of cas phase H/D exchange reactions of protonated glycine oligomers with D2O, CD3OD, CD3CO2D, and ND3. J Am Chem Soc 117, 12840–12854 (1995).

[b45] Jacrot B. Study of Biological Structures by Neutron-Scattering from Solution. Rep Prog Phys 39, 911–953 (1976).

[b46] Svergun D. I., Richard S., Koch M. H. J., Sayers Z., Kuprin S. & Zaccai G. Protein hydration in solution: Experimental observation by x-ray and neutron scattering. Proceedings of the National Academy of Sciences 95, 2267–2272 (1998). 10.1073/pnas.95.5.2267 PMC19315 9482874

[b47] Zhang J. D., Chi Q. J., Dong S. J. & Wang E. K. *In situ* electrochemical scanning tunnelling microscopy investigation of structure for horseradish peroxidase and its electrocatalytic property. Bioelectroch Bioener 39, 267–274 (1996).

[b48] Wu H. . Size-dependent tuning of horseradish peroxidase bioreactivity by gold nanoparticles. Nanoscale 7, 4505–4513 (2015). 25684572 10.1039/c4nr07056a

